# Mixture Models for Distance Sampling Detection Functions

**DOI:** 10.1371/journal.pone.0118726

**Published:** 2015-03-20

**Authors:** David L. Miller, Len Thomas

**Affiliations:** Centre for Research into Ecological and Environmental Modelling, and School of Mathematics and Statistics, University of St Andrews, St Andrews, Scotland, United Kingdom; CEFE, UNITED STATES

## Abstract

We present a new class of models for the detection function in distance sampling surveys of wildlife populations, based on finite mixtures of simple parametric key functions such as the half-normal. The models share many of the features of the widely-used “key function plus series adjustment” (K+A) formulation: they are flexible, produce plausible shapes with a small number of parameters, allow incorporation of covariates in addition to distance and can be fitted using maximum likelihood. One important advantage over the K+A approach is that the mixtures are automatically monotonic non-increasing and non-negative, so constrained optimization is not required to ensure distance sampling assumptions are honoured. We compare the mixture formulation to the K+A approach using simulations to evaluate its applicability in a wide set of challenging situations. We also re-analyze four previously problematic real-world case studies. We find mixtures outperform K+A methods in many cases, particularly spiked line transect data (i.e., where detectability drops rapidly at small distances) and larger sample sizes. We recommend that current standard model selection methods for distance sampling detection functions are extended to include mixture models in the candidate set.

## Introduction

Distance sampling [1, 2] is a suite of methods for estimating the size or density of biological populations. There are two main variants: line and point transects. In both, an observer visits a randomly-located set of transect lines or points and records the distance, *y*, from the transect to each object of interest (i.e., animals or plants of the target species) that is detected within some truncation distance *w* (after which no observation is recorded; truncation may be chosen after the survey has taken place, see Buckland et al [1] for further discussion). Not all objects within *w* are assumed to be detected; instead the observed distances are used to estimate the parameter vector, ***θ***, of a detection function model, *g*(*y*; ***θ***), which describes how the probability of detection declines with increasing distance. An assumption of the conventional method is that *g*(0; ***θ***) = 1. Given an estimate of ***θ***, it is straightforward to estimate population size or density (see below).

A key part of distance sampling, therefore, is specification of the detection function model. Buckland et al. (Chapter 2) [1] provide a set of criteria for judging the utility of candidate model classes. Detection function models should be:

1. flexible, so that they can take a wide variety of shapes;
2. efficient, in the sense that many plausible shapes can be represented using few parameters;
3. flat at zero distance (i.e., *g*′(0; ***θ***) = 0), indicating that objects in the immediate vicinity of the observer are equally detectable; and,
4. monotonic non-increasing with increasing distance (i.e., *g*′(*y*; ***θ***) ≤ 0 for 0 < *y* ≤ *w*), as it is typically unrealistic for objects to become more detectable with increasing distance.

The semiparametric “key function plus series adjustment” (K+A) modelling approach developed by Buckland [3] has become by far the most popular in practice, partly due to its inclusion in the industry-standard distance sampling analysis software Distance [4] and the R [5] package mrds [6] (available on the Comprehensive R Archive Network, CRAN; http://cran.r-project.org/). However, as we demonstrate below, the approach has some drawbacks; in particular, although it meets criteria 1–3, it does not necessarily meet the 4^th^. Our purpose in this article is to propose an alternative class of models, based on mixtures, that meet all 4 criteria and to evaluate its utility.

The approach of Buckland [3] was extended by Marques and Buckland [7] to allow covariates in addition to distance to be included in the detection function, and, for maximum generality, it is this K+A formulation that we describe here. The detection function is thus denoted *g*(*y*, **z**; ***θ***) where **z** is an observation-specific vector of covariates; the formulation of Buckland [3] is simply a special case of this model where there are no additional covariates.

In Marques and Buckland [7], the detection function is modelled as a parametric key function *k* and series expansion *s* of even functions (known as *adjustment terms*) with some parameters ***θ***. *g* is then written as:

$$\begin{array}{c} g\left(y,z;\theta \right)=\frac{k(y,z;\theta )\{1+s(y,z;\theta )\}}{k(0,z;\theta )\{1+s(0,z;\theta )\}}, \end{array}$$

where *k* may be a half-normal, hazard-rate or uniform function and *s* may be zero (i.e., there are no adjustment terms), cosine, simple even polynomial or Hermite polynomial series (though note a uniform detection function may not include covariates). The denominator ensures that detection function evaluates to 1 at zero distance (i.e., *g*(0, **z**; ***θ***) = 1). Model parameters are estimated using maximum likelihood. The recommended strategy for most situations is to choose a small set of key function and adjustment combinations, and for each combination to choose the number of adjustment terms using forward selection, i.e., start with no adjustment terms and fit an increasing number of terms, stopping when the Akaike Information Criterion (AIC) fails to decrease [4]. The combination with the lowest AIC is then selected as the best model. This strategy works well in practice in many cases: the key functions cover a range of realistic shapes for the detection function, so that often zero or one adjustments are sufficient to provide a good fit to the data, resulting in flexible and yet efficient estimation.

The resulting detection functions are capable of being flat at zero distance and the key functions are non-increasing. However, adding adjustment terms can result in non-monotonic functions. Further, when both covariates and adjustments are included in the model the range of the resulting detection function may not be [0, 1]. When there are no additional covariates, one solution is to use constrained maximization, e.g. taking *M* equally spaced distances *y*_1_ = 0, …, *y*_*M*_ = *w* and ensuring that $g(y_{i};\hat{\text{θ}})\geq g(y_{i+1};\hat{\text{θ}})$ and that $g(y_{i+1};\hat{\text{θ}})\geq 0$ for *i* = 1, …, *M* − 1. In Distance this constraint is implemented using the NLPQL routine [8] and in the R package mrds, the SOLNP algorithm [9] is used.

**Fig 1:**
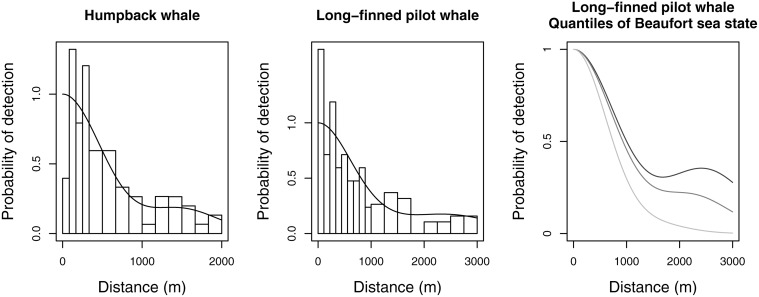
Two examples of detection functions that are not monotone, fitted using conventional key function plus adjustment methods in the software Distance. The left panel shows data from humpback whale: a half-normal detection function with cosine adjustments was selected by AIC [20] but even with constraints in place the detection function is non-monotonic, with a small secondary peak at approx. 1500m. The second and third panels show data and models fitted to long-finned pilot whale where a half-normal detection function was selected with cosine adjustments and Beaufort sea state as a covariate [10]. Due to the inclusion of covariates, no monotonicity constraints could be employed. The middle panel shows the detection function averaged over the covariate values and the right panel the marginal detection function for 25th, 50th and 75th quantiles of the Beaufort sea state covariate; non-monotonicity occurs at approx. 2500m.

A constrained optimisation solution presents a number of problems. First, constrained maximization is a more complex optimization problem than unconstrained maximization; this means that in practice optimization algorithms may fail to find the constrained maximum. Second, constrained maximum likelihood estimates do not have the same appealing properties as their unconstrained relatives—for example the usual estimator of the standard error of the parameters (square root of the inverse of the information matrix) can be biased. Third, constraints can only be applied at a finite number of points (*M* = 10 is used in Distance and *M* = 20 in mrds by default), which can lead to the constraint points missing non-monotonic parts of the function. Though increasing the number of points is an option, this incurs additional computational cost. An example of constrained maximisation failing is shown in the left panel of Fig. 1. Finally, it is not clear how to implement the constraints in the case where there are additional covariates, particularly continuous covariates. One computationally expensive option would be to apply the constraints at every observed covariate combination (at present both Distance and mrds use unconstrained optimization when additional covariates are in the model). The central and right panels of Fig. 1 from Pike et al. [10] show an example of covariate models fitted using unconstrained optimisation: a strongly non-monotonic function has been fitted for some covariate values. Detection probability estimates outside the range [0, 1] are sometimes encountered during maximization when models include covariates. Given the above issues, it seems appealing to use a formulation that guarantees monotonicity from the outset.

Mixture models have been applied in the capture-recapture literature [11–14]. The main utility of mixture models in capture-recapture is in better accounting for between-individual heterogeneity, which can cause severe bias if unmodelled [15]. Unmodelled heterogeneity is not generally considered an issue in distance sampling, provided that detection at zero distance is certain, heterogeneity is not extreme and a flexible detection function model is used ([2], Section 11.12). Mixture models come in two variations: finite (consisting of discrete components) and continuous (infinitely many components, amounting to an integral with a weight function); we consider only finite mixtures here (see Discussion for further elaboration). Finite mixture models offer the potential for flexible modelling since the individual parts of the mixture model (the *mixture components*) can be combined to obtain flexible detection functions, and provided each component is monotonic non-increasing, the resulting combination will also be monotonic non-increasing. In addition, mixture models are potentially well suited to deal with highly heterogeneous detection probabilities, where some part of the population is only observable at close distances while others are readily detected almost regardless of distance (for example bird species where males are more vocal than females). Such a situation results in a “spiked” detection function with a long flat tail—Fig. 1 shows relatively mild examples. In a mixture model, different parts of the sample could be represented by different components, providing a good fit to spiked data and an appealing conceptual explanation for the underlying data.

Here we introduce a new class of distance sampling detection function models, based on mixtures of simple parametric key functions. In the next section, we describe the models. We then illustrate their use and explore their performance. First we investigate performance in terms of the accuracy of estimation of the average probability of detection when data are simulated from a variety of situations. We then go on to investigate survey data from a number of studies that are potentially problematic. We compare results from mixture models with those obtained from the current standard K+A approach, and by using a combined approach where both the mixtures and K+A models are fitted and a final model selected using AIC. An R package, mmds [16] (Mixture Model Distance Sampling), implementing the methods is available from CRAN.

## Methods

### Finite mixture model detection functions: Formulation

Denoting the detection function as *g*, we consider a sum of *J* mixture components *g*_*j*_, scaled by some mixture proportions *ϕ*_*j*_:

$$\begin{array}{c} g\left(y,z;\theta ,\phi \right)=\sum_{j=1}^{J}\phi_{j}g_{j}\left(y,z;\theta_{j}\right), \end{array}$$

where $\sum_{j=1}^{J}\phi_{j}=1$. The distance is denoted *y*, the ***θ***_*j*_s are vectors of parameters for function *g*_*j*_, ***θ*** is a vector of all of the ***θ***_*j*_s, ϕ is a *J*-vector (i.e., vector of length *J*) of all of the *ϕ*_*j*_s, and **z** is a *K*-vector of the associated covariates.

Although other monotonic functions such as hazard-rate could be chosen, and the *g*_*j*_s need not all have the same form, here we let the *g*_*j*_s be half-normal functions:

$$\begin{array}{c} g\left(y,z;\theta ,\phi \right)=\sum_{j=1}^{J}\phi_{j}\exp\left(-\frac{y^{2}}{2\sigma_{j}{\left(z\right)}^{2}}\right). \end{array}$$

Although each mixture component has a different scale, the covariates affect the scale parameters in the same way (though other, more complex, models may be possible).

Covariates are included as in Marques and Buckland [7], by decomposing the scale parameter *σ* (see also Marques et al. [17]). Using *i* to subscript each observation, our formulation for the scale parameter *σ*_*ij*_, is

$$\begin{array}{c} \sigma_{ij}=\exp\left(\beta_{0j}+\sum_{k=1}^{K}\beta_{k}z_{ik}\right), \end{array}$$

where *z*_*ik*_ is the *k* ^th^ covariate for the *i* ^th^ observation. In this case ***θ*** will contain the *β*_0*j*_s and *β*_*k*_s.

We can write the pdf of the observed distances conditional on the observed covariates as [2]:

$$\begin{array}{c} f\left(y|z;\theta ,\phi \right)=\frac{\pi (y)g(y,z;\theta ,\phi )}{\int_{0}^{w}\pi \left(t\right)g\left(t,z;\theta ,\phi \right)\text{d}t}. \end{array}$$

where *π*(*y*) is the pdf of object distances (observed and unobserved). The likelihood can then be formed by taking product of these pdfs over the *n* observations. The specific form of the likelihood differs between line and point transects, because sampler geometry means that the form of *π*(*y*) is different for lines and points. For line transects, with random line placement, we expect an equal number of objects at all distances from the line, and hence *π*(*y*) = 1/*w* (where *w* is again the truncation distance). The likelihood is then given by:

$$\begin{array}{cc} L(\theta ,\phi ;y|z_{1},\ldots ,z_{n}) & =\prod_{i=1}^{n}f\left(y_{i}|z_{i};\theta ,\phi \right)\\ & =\prod_{i=1}^{n}\frac{g(y_{i},z_{i};\theta ,\phi )}{\mu_{i}\left(z_{i}\right)}\\ & =\prod_{i=1}^{n}\frac{\sum_{j=1}^{J}\phi_{j}g_{j}\left(y_{i},z_{i};\theta_{j}\right)}{\mu_{i}\left(z_{i}\right)} \end{array}$$

where *μ*_*i*_(**z**_*i*_), the *effective strip width* (for covariate combination **z**_*i*_), is given by:

$$\begin{array}{c} \mu_{i}\left(z_{i}\right)=\sum_{j=1}^{J}\phi_{j}\int_{0}^{w}g_{j}\left(y,z_{i};\theta_{j}\right)\text{d}y. \end{array}$$ (1)

For point transects, with random point placement, the expected number of objects increases with increasing distance from the point, and hence *π*(*y*) = 2*y*/*w* ^2^, giving

$$\begin{array}{cc} L(\theta ,\phi ;y|z_{1},\ldots ,z_{n}) & =\prod_{i=1}^{n}f\left(y_{i}|z_{i};\theta ,\phi \right)\\ & =\prod_{i=1}^{n}\frac{2\pi y_{i}g\left(y_{i},z_{i};\theta ,\phi \right)}{\nu_{i}}\\ & =\prod_{i=1}^{n}\frac{2\pi y_{i}\sum_{j=1}^{J}\phi_{j}g_{j}\left(y_{i},z_{i};\theta_{j}\right)}{\nu_{i}} \end{array}$$

where *ν*_*i*_, the *effective area of detection* (for covariate combination **z**_*i*_), is defined as:

$$\begin{array}{c} \nu_{i}=2\pi \sum_{j=1}^{J}\phi_{j}\int_{0}^{w}yg_{j}\left(y,z_{i};\theta_{j}\right)\text{d}y. \end{array}$$ (2)

For both line and point transects, parameters are estimated using maximum likelihood. Practicalities associated with this maximization, along with analytic derivatives of the likelihood are described in S1 Appendix and S1 Text. The best number of mixture components to use for inference can be determined using standard model selection techniques, such as Akaike’s Information Criterion (AIC), and goodness-of-fit of fitted models can be assessed just as for K+A models using, for example quantile-quantile plots and Kolmogorov-Smironov tests (see Buckland et al. [2], Section 11.11).

In this article, we assume the distance data are in the form of “exact” object-transect distances; alternatively, distances can be grouped into intervals, with pre-defined cutpoints (e.g., 0–10m, 10–20m, etc.), so that the data are the distance interval of each observation. In this case, a multinomial likelihood is obtained (see, e.g. Buckland et al. [1], Section 3.3.2). Also, in some cases (e.g., some aerial surveys), objects below a defined distance are not counted—so-called “left truncation” ([1] Section 4.3.2). The likelihood is readily amended to account for this, by changing the lower limit of integration in equation (1) or (2).

### Estimating population size

Population size can be estimated using the Horvitz-Thompson-like estimator [7]:

$$\begin{array}{c} \hat{N}=\frac{A}{a}\sum_{i=1}^{n}\frac{1}{{\hat{p}}_{i}} \end{array}$$ (3)

where *A* is the area of the study region for which population size is being estimated, *a* is the size of the sampled area, and *p*_*i*_ is the probability of the *i* ^th^ observation being detected given it is within the sampled area. For line transects, *a* = 2*wL* where *L* is the total line length, and

$$\begin{array}{c} {\hat{p}}_{i}=\frac{1}{w}\sum_{j=1}^{J}{\hat{\phi }}_{j}\int_{0}^{w}g_{j}\left(y,z_{i};{\hat{\theta }}_{j}\right)\text{d}y. \end{array}$$

For point transects, *a* = *πw* ^2^ *k* where *k* is the number of points, and

$$\begin{array}{c} {\hat{p}}_{i}=\frac{2\pi }{w^{2}}\sum_{j=1}^{J}{\hat{\phi }}_{j}\int_{0}^{w}yg_{j}\left(y,z_{i};{\hat{\theta }}_{j}\right)\text{d}y. \end{array}$$

A standard summary statistic is the average detection probability for an animal within the sampled area, *P̂*_*a*_, which is given by:

$$\begin{array}{c} \hat{P_{a}}=n/\hat{N}. \end{array}$$

Estimators for the variances of *N̂* and *P̂*_*a*_ are given in S2 Text.

### Examples

#### Simulated data

We wish to ensure that the class of models we propose can be applied to a wide variety of situations that may arise. Extensive simulations were therefore carried out to investigate performance (in terms of the accuracy of estimation of *P*_*a*_) when the true detection function model is not known to the estimation procedure. The average detection probability, *P*_*a*_, is related to the estimated abundance as seen above and is easily calculated as a simple statistic to summarise and compare the fitted models.

Buckland et al. [1] show that accurate results are readily obtained in situations where there is a wide “shoulder” of high detection probability at small and medium distances: in such situations, the dependence on having a good detection function model is only slight. Hence, we focus here on a variety of more challenging scenarios. We generated data from commonly used detection function models (half-normal and hazard-rate [1], as well as exponential power series [18]), though with parameters that lead to more challenging estimation problems.

Each simulation involved generating 200 replicate datasets from a specified detection function model (assuming the entire study area was included within the surveyed transects, i.e., *A* = *a* in equation (3), and a truncation distance of *w* = 1), fitting each dataset with a range of mixture and key series plus series adjustment (K+A) models, and in each case recording estimated parameter values and abundance from the model with the lowest AIC in each of: mixture models, K+A models, and both combined. Mixture models with 1-, 2-, and 3-point half-normal components were fitted to the data along with two K+A models: half-normal plus cosine adjustments and hazard rate plus simple polynomial adjustments, both with monotonicity constraints implemented as described above and with a maximum of 3 adjustments. Mixture models and K+A models were fitted using the R packages mmds (version 1.1) and Distance [19] (a simplified interface to mrds; version 0.6.1) respectively, both written by the authors.

**Fig 2:**
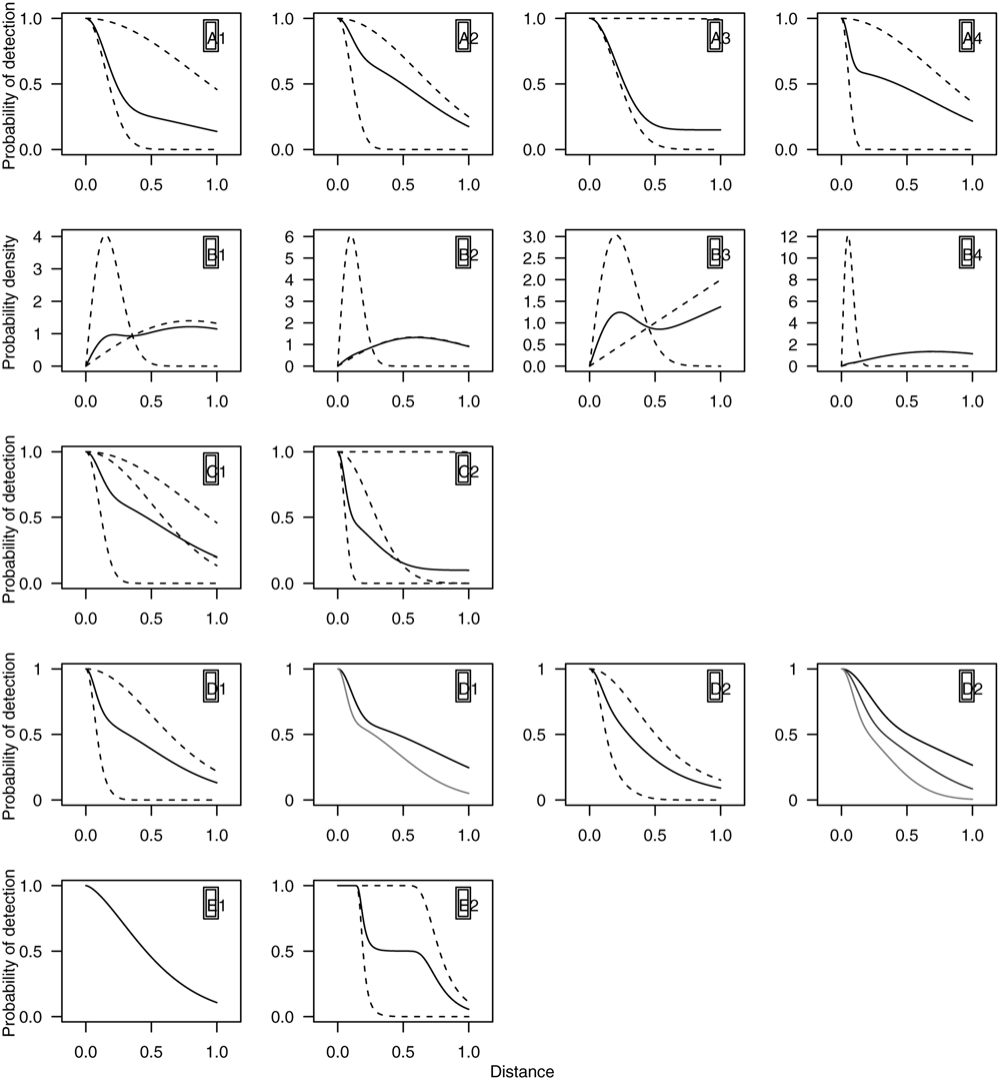
Plots of the models used in the simulation. Group A (top row): detection functions for four line transect senarios with no covariates (solid lines) and their constituent mixture components (dashed lines). Group B (second row): pdfs for four point transect simulations with no covariates (solid lines), with associated component pdfs (dashed lines), rescaled so the area under each curve is one; the detection functions are as in the top row. Group C (third row): two 3-point mixture scenarios for non-covariate line transect data, again with their constituent mixture components (dashed lines). Group D (fourth row): two covariate model scenarios, the first two panels are for a binary covariate scenario, the second two for a continuous covariate scenario; first panels in each pair show the detection function averaged over the covariates (along with the mixture components, similarly averaged) and the second panels show marginal detection functions with the levels (or quartiles) of the detection function. Group E (fifth row): exponential power series model and a 2-point mixture of hazard-rate function (see S2 Appendix for formulation) for two line transect scenarios.

Fourteen different simulation scenarios were investigated, in five groups, as described below and illustrated in Fig. 2, one line per group. True parameter values and summary statistics are given in S2 Appendix. For each scenario, a simulation was performed at each of five sample sizes (number of observations, *n*): 30 (low), 60 (recommended minimum for line transects [1]), 120 (adequate), 480 (large) and 960 (very large). We anticipated performance would depend upon sample size, because: the methods are likelihood-based and hence only asymptotically unbiased even if the correct model is fitted; the use of AIC to select model complexity meant that more flexible (and hence accurate) models could be expected to be selected given larger sample sizes. Mixture models are “parameter hungry” compared with K+A models, in the sense that each additional mixture component requires 2 extra parameters, while each additional adjustment term requires only one and hence, given the use of AIC for model selection, the relative performance of the two approaches may change at different sample sizes.

*Group A. Line transect with 2-point half-normal mixture detection functions*. Four scenarios were tested, representing a range of potentially challenging detection functions. Scenarios A1 and A2 both have mixture components with quite different scale parameters, but in A1 the majority of data come from the less detectable component while in A2 it comes from the more detectable component. A3 tests the behaviour of the models when the scale parameter of one of the mixture components is very large relative to the truncation distance. One component of A4 is a large spike (i.e., a sharp decline in detectability at small distances) in comparison to the other component leading to high heterogeneity in detection probability, which is similar to some of the data we analyse in the case studies, below.

*Group B. Point transect with detection functions as in the previous scenario*. The geometry of point transect sampling means there are few animals close to the point relative to those at larger distances. Hence, for a given sample size of observations, there are far fewer at small distances than for line transects, making it harder to accurately model the detection function in the critical region close to the point. We therefore anticipate that performance will be worse for point transects. For this group, Fig. 2 shows pdfs of the observed distances.

*Group C. Line transect with 3-point half-normal detection functions*. Two scenarios were tested. C1 has a detection function much like A2, enabling us to investigate the efficacy of model selection (i.e., we expect a 2-point mixture to be selected and to produce good results). C2 is a more complex shape that could only be created using a 3-point mixture; in particular (as with A3), one of the components has a large scale parameter relative to the truncation distance.

*Group D. Line transect with 2-point half-normal detection functions, and additional covariates*. We used covariate models to test two aspects of model robustness. In the first, we assumed the covariate values were observed, and included covariate models in the candidate set, along with distance-only models. Our prediction was that (at large sample sizes at least) covariate models would be selected and estimation of *P*_*a*_ unbiased. In the second, we assumed the covariate values were not observed, and hence covariate models were not in the candidate set. Our expectation was that (at larger sample sizes) more complex mixture distributions would be selected to compensate for the additional unobserved complexity, and that estimation of *P*_*a*_ would not be greatly affected. Two scenarios were tested. D1 had a binary factor covariate, with half the observations having one covariate value and half the other. D2 had a continuous covariate, whose fixed values were generated from a standard normal distribution function. Detection functions are shown in the fourth row of Fig. 2, along with the marginal detection functions for the levels/quartiles of the covariates. Note that, for the unobserved covariate models, D1 is equivalent to a 4-point mixture, while D2 is equivalent to a 2-point continuous mixture; neither of these models were in the candidate model set. In the case of the K+A models, and in line with common practice, if covariates were included in the models then adjustment terms were not.

*Group E. Line transect with other detection functions*. The above models all use the same functional form for *g*_*j*_ in generation and fitting. Here we tested the model robustness using two alternative data generating functions, not in the candidate model set (see S2 Appendix for formulation). E1 used an exponential power series function (a generalization of the half-normal function with an additional shape parameter); E2 used a mixture of two hazard-rate functions, giving a shape that may be difficult to fit with half-normal models.

#### Case studies

The first two case studies return to the datasets depicted in Fig. 1. The left panel of Fig. 1 show clear non-monotonicity, which we wish to address with our mixture detection functions. This first case study also includes two other species, illustrating how the new approach can fit survey data as well as, or better than, the K+A approach when there are not issues of monotonicity. In the second case study covariates cause the non-monotonicity (seen in the right panel of Fig. 1), which we can also address within our mixture model framework. The third case study demonstrates modelling of spiked line transect data (of wood ants), for which the mixtures may yield more flexible models than K+A methods. Finally, the fourth example is a large point transect dataset (of Hawaiian amakihi), which include covariates.

#### Case study: British Columbia marine mammals

Williams and Thomas [20] used a data from a line transect survey to study several species of marine mammal off the coast of British Columbia, Canada. Here, we investigate three species: harbour seal (*Phoca vitulina*) in water (the data also contained observations of hauled-out animals, which were not analysed here), harbour porpoise (*Phocoena phocoena*) and humpback whale (*Megaptera novaeangliae*). Truncation distances were set at 500m, 500m and 2000m for each species respectively, giving sample sizes of 232, 59, and 70 observations.

#### Case study: Long-finned pilot whales

Pike et al. [10] analyzed observations of 84 pods of long-finned pilot whales (*Globicephala melas*), sighted as part of a line transect survey, the North Atlantic Sightings Survey NASS-2001. The Beaufort sea state was recorded as a covariate during the survey and enters the authors’ model as either a continuous variable, or a factor with 2 levels (0–1, 2+), 3 levels (0–1, 2, 3+), or 5 levels (0, 1, 2, 3, 4, with one value of 3.5 coded as 4).

#### Case study: Wood ants

Borkin et al. [20] analyse data on two species of wood ant (*Formica aquilonia* and *Formica lugubris*) collected during a line transect survey of the Abernethy Forest, Scotland, in 2003. The number of nests sighted was 150, with the farthest being 72.04m from the transect, although 45% of the nest sightings lay within 4m of the line. As part of their analysis, several different truncation distances were used. Larger truncation distances led to a large variance in the encounter rate estimates and hence in overall abundance estimates [21]. This is due to the spike caused by the large number of detections close to the line (see S4 Fig). As well as distances, three covariates were recorded: habitat type (a four level factor), the size of each nest (a continuous variable, calculated as half-width multiplied by height) and species (a two level factor).

#### Case study: Amakihi

Marques et al. [17] analyse point transect data on a Hawaiian songbird, the Amakihi (*Hemignathus virens*). The data consist of 1243 observations (after truncation at 82.5m), collected at 41 points between 1992 and 1995, together with three covariates in addition to distance: the observer (a three level factor), minutes after sunrise (continuous) and hours after sunrise (a six level factor).

## Results

### Simulation results

**Fig 3:**
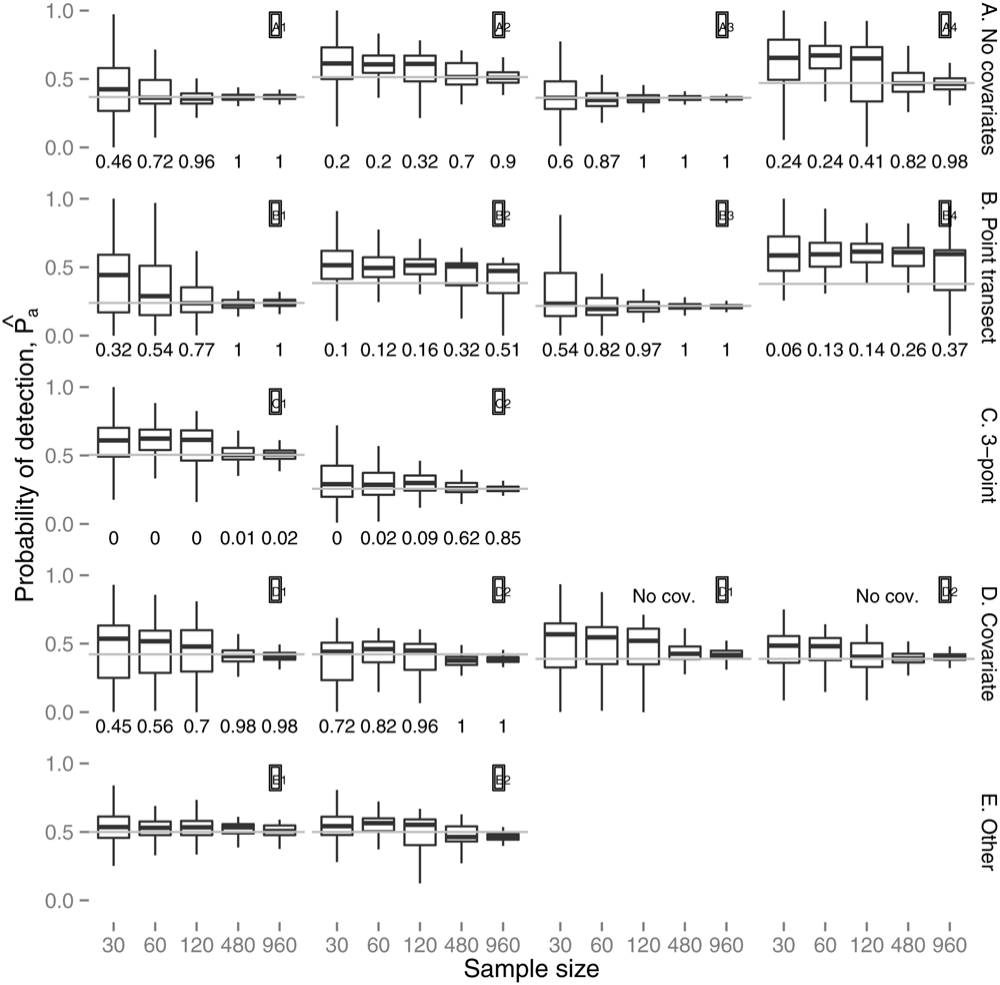
Simulation results: boxplots of the estimated average detection probabilities, *P*_*a*_, for the best mixture model (by AIC score). Layout is as in Fig. 2. Grey lines indicate the true value of the average detection probability. Numbers underneath each boxplot give the proportion of AIC best models that were of the same form as the model that the data was simulated from (e.g., Scenario D1 the proportion of AIC best models that were 2-point mixtures that included the covariate in the model).

Fig. 3 summarizes the estimates of *P*_*a*_ obtained if the candidate model set contains only mixture models (including 1-point mixtures); the numbers below each boxplot are the proportion of times the model selected by AIC was the model which generated the data. S1 Fig shows the distribution of estimates when only K+A models are used, giving a baseline to compare the mixture model results against (S2 Fig). Results using the recommended modelling strategy of both mixture and K+A models is shown in S2 Fig, and the number of times each model is chosen using the combined modelling strategy is shown in S3 Fig.

For Group A, the mixture approach produced unbiased results for scenarios A1 and A3 at all but the lowest sample size; even for the *n* = 30 scenarios the bias was small, despite the correct 2-point mixture model being selected only 46–60% of the time (Fig. 3—the half normal model was selected the remainder of the time). The K+A approach also performed well (S1 Fig). Unsurprisingly, therefore, the combined approach performed well (S2 Fig); what was a little surprising was that the correct model was only selected 60–76% of the time at the highest sample sizes for scenario A1, with the hazard-rate K+A model selected the remainder (S3 Fig). Scenarios A2 and A4 showed positive bias at smaller sample sizes under the mixture approach; bias reduced substantially by 480 observations, where a large proportion of the selected models were 2-point mixtures. Unlike scenarios A1 and A3, the detection functions in scenarios A2 and A4 were evidently not well approximated by a half-normal, and hence at lower sample sizes where the two point mixture tended not to be selected, the results were biased. The K+A approach did not fare well with these scenarios, showing strong positive bias even at the largest sample sizes. In combination, the mixture models were chosen over K+A models at larger sample sizes, and so the combined modelling approach produced much better results than K+A alone.

As expected, results were worse for the point transect scenarios of Group B. Estimates from the mixture approach were biased at low sample sizes for B1, when the two-point model was rarely selected, but were unbiased given 120 observations and greater. Estimates for B3 were unbiased. For B2 and B4, results were positively biased at small sample sizes, just as with A2 and A4, but unlike the line transect scenarios the bias did not disappear even at the largest sample size. This is unsurprising given the very small number of detections coming from the less detectable mixture component (see Fig. 2—the marginal pdf is almost identical to that of the easier to detect mixture component). Bias was generally worse with the K+A approach (S1 Fig), and the combined approach (S2 Fig) produced marginally better results than K+A alone; for scenarios B1 and B3 the combined results were much better than K+A alone.

Group C were the 3-point mixture scenarios. For C1, results were similar to A2—unsurprising, given the similarity in detection functions. A 3-point mixture model was almost never chosen by AIC (Fig. 3). For C2, estimates were surprisingly good, even when the 3-point mixture was not the selected model, at lower sample sizes. Evidently, the function is well approximated by a 2-point mixture, although at larger sample sizes (*n* = 480 and above), the 3-point model is preferred by AIC. In both cases, the K+A results were worse (S1 Fig), although they were not far from unbiased for C2. In the combined results, the mixture models were chosen most (52–68%) of the time for model C1, while for C2 the mixture models were chosen less often (15–60% of the time); despite this, the results were just as good as those using mixtures alone (S2 Fig).

We first address the results of the Group D simulations when covariates were available for inclusion in candidate models. Results for D1 were positively biased at lower sample sizes, but less so as the sample size increased, and almost unbiased by 120 observations, where the correct model was selected most of the time (Fig. 3). Results for D2 were close to unbiased at all sample sizes. Estimates from the K+A models were positively biased at almost all sample sizes for D1, and almost unbiased for D2 (S1 Fig).

When covariate information is not available for fitting the model, the mixture model detection functions still performed well, showing that when covariates are not available mixture components can compensate, though not through using additional components (see S3 Fig, 3-point mixtures are never AIC-best models). For the K+A models without covariates, performance was also similar to that from the covariate models, indicating that the flexibility provided by the series adjustment can compensate for lack of covariate information in that framework. However, results were still slightly biased even at large sample sizes (S1 Fig). As might be expected, bias was less when both approaches were combined (S2 Fig).

The Group E results were encouraging. Although the mixture formulation was biased even at large sample sizes, the bias was always small (Fig. 3), and generally no worse than that under the K+A formulation, which also showed a small bias (S1 Fig). We had anticipated good performance of the mixture models for scenario E1, since the detection function shape is not far from half-normal; however it was not obvious that performance would be good for E2, where the marginal shape cannot be approximated well by a mixture of half-normal functions. The combined strategy was no worse than either formulation alone in terms of bias.

### Case studies results

#### British Columbia marine mammals

**Fig 4:**
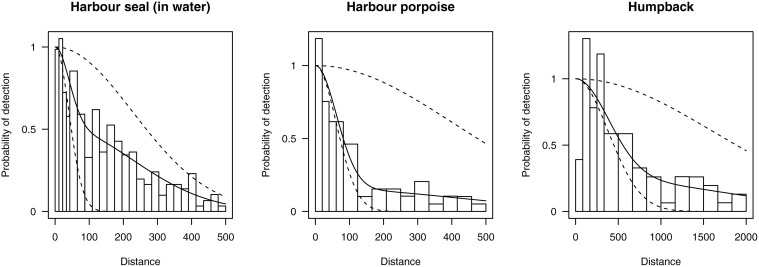
Plots of the mixture model detection functions fit to the British Columbia marine mammal data. In each case the best mixture model by AIC was a 2-point mixture. Dashed lines show the mixture components.

Results are summarised in Table 1 and detection functions for the AIC-best models are shown in Fig. 4. In each case mixture models were two component models. For harbour seal, the mixture model had a lower AIC than for the K+A model reported in Williams and Thomas [20]. The mixture model *P̂*_*a*_ is approximately 20% lower, implying that the previous estimate of *N̂* may have been an underestimate (as *P*_*a*_ decreases, 1/*P*_*a*_ increases in the Horvitz-Thompson estimator giving a larger estimate of abundance). For harbour porpoise, the mixture model AIC is almost 1.5 points higher than the K+A model, which was a hazard-rate with no adjustments. Hence, the model likelihoods are very similar, but the penalty due to the 2-point mixture having an additional parameter prevents it from being selected. The *P̂*_*a*_ from the two models are very close. Lastly, for humpback whales, the mixture model AIC is almost 3 points higher than the K+A model—however, one advantage of the mixture model is that the fitted function is monotone (Fig. 4) while the K+A function is not (Fig. 1). Again, the estimated *P̂*_*a*_s are very similar.

**Table 1:** Comparison of case study analysis results.

**Species**	**Model**	**ΔAIC**	*P̂*_*a*_	*%CV P̂*_*a*_	**K-S *p***
Harbour seal (in water)	Hn + cos(2)	1.19	0.425	7.55	0.52
	Hn 2-pt	0.00	0.335	15.38	0.94
Harbour porpoise	Hr	0.00	0.212	32.0	0.99
	Hn 2-pt	1.43	0.254	18.18	0.99
Humpback whale	Hn + cos(2)	0.00	0.386	12.64	0.67
	Hn 2-pt	2.88	0.381	18.48	0.64
Long-finned pilot whales	Hn + cos(2) BSS (cont.)	1.94	0.452	8.69	0.48
	Hn 2-pt BSS (cont.)	0.00	0.216	24.17	0.67
	Hn 2-pt BSS5	0.29	0.208	28.84	0.82
	Hn 2-pt BSS2	0.43	0.211	23.39	0.95
	Hn 2-pt BSS3	11.71	0.270	17.46	0.99
	Hn 2-pt	13.86	0.295	17.17	0.95
Wood ants	Hr nest.size + habitat	6.29	0.195	21.72	0.89
	Hn 2-pt habitat + nest.size	0.00	0.179	17.55	0.72
	Hn 2-pt nest.size + species + habitat	1.81	0.178	18.09	0.83
	Hn 2-pt nest.size	4.37	0.214	15.19	0.76
	Hn 2-pt nest.size + species	4.65	0.210	15.84	0.77
	Hn 2-pt habitat	14.00	0.188	14.85	0.97
	Hn 2-pt habitat + species	15.96	0.186	14.94	0.99
	Hn 2-pt None	17.34	0.184	15.46	0.96
	Hn 2-pt species	19.32	0.184	15.48	0.94
Amakihi	Hr obs + mas	0.00	0.319	5.11	0.08
	Hn 2-pt obs+mas	0.69	0.279	6.1	0.14
	Hn 2-pt obs	1.31	0.279	5.86	0.04
	Hn 2-pt obs+has	5.15	0.283	6.21	0.23
	Hn 2-pt mas+has+obs	7.12	0.282	6.33	0.35
	Hn 2-pt mas	27.73	0.284	6.52	0.31
	Hn 2-pt None	28.10	0.283	6.21	0.12
	Hn 2-pt has	29.81	0.282	6.95	0.33
	Hn 2-pt mas+has	31.79	0.282	6.97	0.43

Comparison of results from Williams and Thomas [20], Pike et al. [10], Borkin et al. [21] and Marques et al. [17] with results from fitting mixture model detection functions. Table columns give case study species, model fitted (In the table “(cont.)” denotes that the covariate was included in the model as continuous, otherwise covariates entered the model as factors; BBSn indicates Beaufort sea state with *n* factors); cos(*x*) indicates a Cosine adjustment of order *x*), difference in AIC from AIC-best model (denotes with ΔAIC = 0), average detectability, percentage coefficient of variation in average detectability and *p*-value from a Kolmogorov-Smirnov goodness-of-fit test. In each case the first line for each data set is the model from the original article and the subsequent models are in ΔAIC order. Mixture model components were selected by AIC (ΔAIC from best model is listed).

#### Long-finned pilot whales

**Fig 5:**
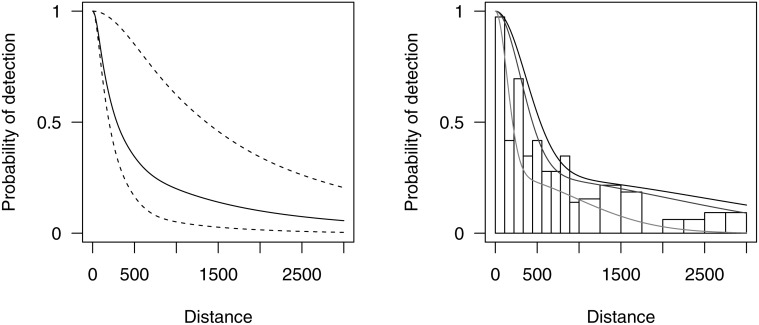
The (AIC) best model for the long-finned pilot whale data: a 2-point mixture model detection function with Beaufort sea state as a continuous covariate. Left: the average detection function (detection function evaluated over the range (0, *w*) for each covariate combination and was then averaged point-wise) with components as dashed lines. Right: the marginal detection function with the quantiles (25%, 50% and 75%) of the Beaufort sea state.

A mixture model detection function was fitted with each covariate, as well as a model with no covariates. The best model by AIC score (Table 1) was a 2-point mixture with Beaufort sea state included as a continuous covariate. Fig. 5 shows the average detection function (in the sense that a detection function was evaluated over the range (0, *w*) for each covariate combination and was then averaged point-wise) and the marginal detection function with the quartiles of Beaufort sea state. None of the non-monotonic behaviour seen in Fig. 1 can occur when a mixture is used.

#### Wood ants

All combinations of main effects were fitted (Table 1), and the best model by AIC was a 2-point mixture with nest size and habitat as covariates (S4 Fig). This model had an AIC that was considerably (6 points) lower than the AIC-best K+A model, a hazard-rate with the same covariates. *P̂*_*a*_ is about 10% lower when estimated using the mixture model.

#### Amakihi

The AIC-best mixture model was a two point mixture with observer and minutes after sunrise as covariates (S5 Fig), closely followed by the model with only observer as a covariate (Table 1). In this case a hazard-rate with observer and minutes after sunrise as covariates performed better than mixtures in AIC terms, although by less than 1 AIC point. The difference in *P̂*_*a*_ between these two models is about 15%. It is encouraging that there is such a small difference in AIC, and that covariate mixture models were selected over mixture models without covariates, despite the large number of parameters that such models entail.

## Discussion

We have investigated and demonstrated the utility of detection functions constructed from mixtures of half-normal functions in both line and point transect distance sampling. We also show that covariates can be readily included in such models. Further, these mixture detection functions can be simply “dropped into” other extensions of conventional distance sampling such as: methods for dealing with incomplete detection at zero distance [22, 23] (for these models, there is an additional mark-recapture component to the likelihood, where mixture models could also be used, as in [11–14]), spatial models for distance sampling data [24, 25] or models for surveys where distances were measured with error [26]. Though we were necessarily limited to only a few example data sets on specific taxa, we note that there is no limitation to the species or survey setup that mixture model detection functions can be used with.

We have shown that the mixture models perform well on both simulated and survey data where traditional methods produce suboptimal results. In many cases the proposed model outperformed K+A models in AIC terms, which is surprising given that the mixture models in question often had more parameters. In particular mixture model detection functions appear useful when dealing with line transect data that has a spike in detection probability at small distances, though we note that it is better to avoid collecting such data in the first place, where possible ([1], p. 42) (spikes are commonly caused by observers spending too much effort near the trackline/point and not looking further afield). Also, other non-detection-related factors can cause a spike, such as rounding of measurements or responsive animal movement, and if present in the data these should be dealt with using other analysis strategies or field methods [1]. For line transect surveys, unbiased estimation of *P*_*a*_ was possible even for very spiked detection functions, so long as the sample size of observations was large (Scenarios A2 and A4). By contrast, estimates remained badly biased at all sample sizes for the equivalent point transect scenarios (B2 and B4). For such surveys, where such a small proportion of the data comes from the closer distances, then perhaps the only effective solution is to constrain the fit, for example using a Bayesian approach with strong priors on the detection function parameters.

We note that in our case studies, a larger coefficient of variation in the average detectability was reported with mixtures than with half-normal K+A models but mixtures seemed to have lower CVs (again, of *P̂*_*a*_) than hazard-rate K+A models (in the line transect case, ignoring non-monotonic K+A models; see Table 1). This can be explained by considering the flexibility of the detection function. A half-normal detection function is relatively inflexible so uncertainty is low (since there is only one parameter and it only affects the scale of the function). However, for a hazard-rate model the shoulder can vary from very small (spiked) to very large (depending on the shape parameter), so the uncertainty in this more flexible model is larger. Mixtures of half-normals lie somewhere in between these two options (only the scales change which are weighted, we are then summing smaller variances).

Simulations show that small sample sizes do not support the use of mixture models with a high number of components, even when the data were generated from such a model. We avoid poorly fitting models of this sort by using both K+A and mixture detection functions and selecting the best between them (comparing Fig. 3 with S1 Fig). This integrated approach is builds upon current model selection procedures for a detection function analysis—currently selection is made between different K+A formulations and number of adjustment terms using AIC; mixture models simply add another alternative detection function where rather than adjustment terms, mixture components are selected. So existing key-only models are special cases of the mixture detection functions.

In simulation we observed that 3-point mixture did not act as good surrogates for missing covariate information; 2-point mixtures were generally chosen by AIC (though these 2-point models performed well at higher sample sizes). In our case studies, 2-point mixtures consistently provided the best fit. Only examination of further data will show whether 3-point and higher mixtures can be supported, however we note that when the K+A series formulation is used, detection functions with 5 or more parameters are rarely selected by AIC (a 3-point mixture with no covariates requires 5 parameters). These results echo those in capture-recapture literature [11], where often only 2 component mixtures were selected.

We have compared the new mixture approach for modelling detection functions with the most widely used alternative, K+A. However, other approaches exist, for example nonparametric and semiparametric kernel estimators (see Eidous [27] and references therein). So far as we are aware, all current alternatives fail some of the criteria given in the introduction—for example, the kernel functions can be non-monotonic. Giammarino & Quatto [28] have proposed a “mixture model” detection function—their model takes a rather different form to the mixtures we describe here (simply exp(−*x* ^2^/(2*σ* ^2^)) − *x*/*τ*)), though their results indicate there is little difference between their model and K+A approaches.

The mixture component used here was a half-normal, but other component functions may prove useful. In particular, a mixture of hazard-rate functions with different shape and/or scale parameters for each component may be better at fitting detection functions with a wide shoulder, a steep drop-off and then a second plateau in detectability (see E2 in Fig. 2, which was generated from a mixture of two hazard-rate functions). Further, a mixture of a half-normal (or hazard-rate) and a uniform kernel may prove useful—this would have only two (or three) parameters, and hence may be more competitive (in AIC terms) with K+A models.

Another potentially useful extension is continuous mixtures of the form

$$\begin{array}{c} g\left(x\right)=\int_{R}\varphi \left(\kappa \right)g_{\kappa }\left(x,Z;\theta ,\kappa \right)\text{d}\kappa \end{array}$$

where *φ*(*κ*) is a weighting function that controls the mixing of *g*_*κ*_. Provided that an appropriate function can be chosen for *φ*, more flexible models could be used whilst keeping the number of parameters low. In addition, a combination of both finite and continuous mixtures could be used, echoing the work in capture-recapture [14]. Such models require more complex optimisation procedures in order to estimate their parameters in a maximum likelihood context, though are well suited to a Bayesian setting.

Mixture model detection functions based on half-normal components are available as an R package, mmds, which is available on CRAN. These models will be added to the next version of the Distance for Windows software and the R package Distance.

## Supporting Information

S1 Appendix Optimization details. (PDF)

S2 Appendix Simulation parameters. (PDF)

S1 Text Derivatives of the likelihood. (PDF)

S2 Text Variance estimation for mixture model detection functions. (PDF)

S1 Fig Simulation results: boxplots of the estimated average detection probabilities, *P*_*a*_, for the best K+A model (by AIC score). Grey lines indicate the true value of the average detection probability.
(EPS)

S2 Fig Simulation results: boxplots of the estimated average detection probabilities, *P*_*a*_, for the best model (by AIC score) for both mixture and K+A models. In each case the best overall model was selected, reflecting the modelling approach undertaken in practice. Grey lines indicate the true value of the average detection probability. Numbers underneath each boxplot give the proportion of AIC best models that were of the same form as the model that the data was simulated from (e.g., in scenario D1, the proportion of AIC best models that were 2-point mixtures that included the covariate in the model). Numbers above each model give the proportion of times that the AIC best model was a 2- or 3-point mixture model.
(EPS)

S3 Fig Simulation results: stacked bar charts showing the number of models selected by AIC that fall into the given model classes. Layout is as in S2 Fig. “hn” is a half-normal detection function (i.e. 1-point mixture) and “hr” is a hazard-rate detection function (no adjustments). K+A indicates a key function plus adjustment term model where “cos” is cosine and “poly” are simple polynomial adjustments. MMDS is a mixture model with 2 or 3 components (“2-pt” or “3-pt”, respectively). “(cov)” indicates that covariates were included in the model.
(EPS)

S4 Fig Plot of the detection functions for the AIC best model for the ants data set (2-point mixture with nest size and habitat as covariates). The first panel shows the average detection function (dashed lines are the two mixture components of the detection function, averaged over covariate values). The second and third panels show the quartiles of nest size and the levels of habitat type respectively.
(EPS)

S5 Fig Plots of the (AIC) best mixture model for the Amakihi data: a 2-point mixture with observer and minutes after sunrise as covariates. Top row: detection function averaged over covariates (dashed lines are each mixture component averaged over covariates), marginal detection function showing the levels of observer (averaged over the values of minutes after sunrise) and marginal detection function for minutes after sunrise ranging between 0 and 300 minutes (averaged over the levels of observer), as in Marques et al (2007) [17]. Bottom row: pdf of distances averaged over the covariate values.
(EPS)
