# Supplementary material for: Mixture Models for Distance Sampling Detection Functions
Source: PLoS One. 2015 Mar 20;10(3):e0118726. doi: 10.1371/journal.pone.0118726 (PMC4368789; doi:10.1371/journal.pone.0118726)
Supplement: S1 Appendix — (PDF) [file pone.0118726.s001.pdf]

Appendix S1: Optimization details

David L. Miller[1,*], Len Thomas[1]
1 School of Mathematics and Statistics, and Centre for Research into Ecological and Environmental Modelling, University of St Andrews, St Andrews KY16 9LZ, Scotland
∗ E-mail: dave@ninepointeightone.net

In practice, maximization is performed on the log-likelihood. However, as noted in the literature [1,2], mixture model likelihoods can be notoriously multimodal. This can cause problems when finding MLEs of the parameters. Here simulated annealing (SANN; [3, Chapter 10]) was used to explore the parameter space (for 500 iterations) then after that the Broyden-Fletcher-Goldfarb-Shanno method (BFGS; [3, Chapter 10]) was used to find the maxima (the implementations in the R function optim() were used). These two steps were run 5 times. This two step approach appears to be satisfactory in most cases. The EM algorithm [4] was tested although there was no significant performance increase (in terms of computational time or parameter precision) over using BFGS with SANN. To aid the optimization, analytic derivatives were also used; these are given in Text S2.

Starting values

Beavers and Ramsay [5] give a method for estimating starting values for the scale parameter of a half-normal detection function. In the non-covariate case, the estimate is given as the intercept parameter from intercept only regression on $\log(y + \frac{w}{1000})$ (where w denotes the truncation distance, as above). For covariate models, the equation used for the σ is used in the regression and the estimated parameters from the linear regression are used as the starting values for the βs.

A similar approach can be use in the mixture case by dividing the sorted distances into J equal parts. For each of these parts a Beavers and Ramsay-type estimate is used for the βs. The mixture weight ϕ_j had starting values of $1/J$ since there is no reason *a priori* to believe anything else.

Parametrisation of the mixture proportions

When using 2-point mixtures, the constraint that the mixture proportions must sum to unity is enforced by definition (since $\phi_2 = 1 - \phi_1$). However, in J-point mixtures when $J > 2$ ensuring that the proportions sum to 1 is not guaranteed. The obvious way to get around this would be to penalise the likelihood, should the optimisation procedure propose values for the ϕ_js that are not in accordance with this condition. This is, of course, inefficient and ugly. Instead, a parametrisation is used for the mixture proportions which yields ϕ_js that comply.

Rather than estimating the ϕ_js, estimate α_ps ($p = 1, \ldots, J$), where the relationship between the two is:

$$\phi_j = F(\sum_{p=1}^{j} e^{\alpha_p}) - F(\sum_{p=1}^{j-1} e^{\alpha_p}) \qquad \text{for } 1 \leq j \leq J-1$$

and

$$\phi_J = 1 - \sum_{j=1}^{J-1} \phi_j$$

where F is any continuous CDF on $(0, \infty]$. Exponentiation ensures that $e^{\alpha_p} \geq 0$, so α_p may lie anywhere on the real line, allowing unconstrained optimisation. Summing these orders the ϕ_js, since only offsets are estimated. Finally, using the cumulative density function ensures that the ϕ_js sum to 1. In practise the Gamma$(3, 2)$ CDF is (somewhat arbitrarily) used. Figure 1 illustrates the relationship.

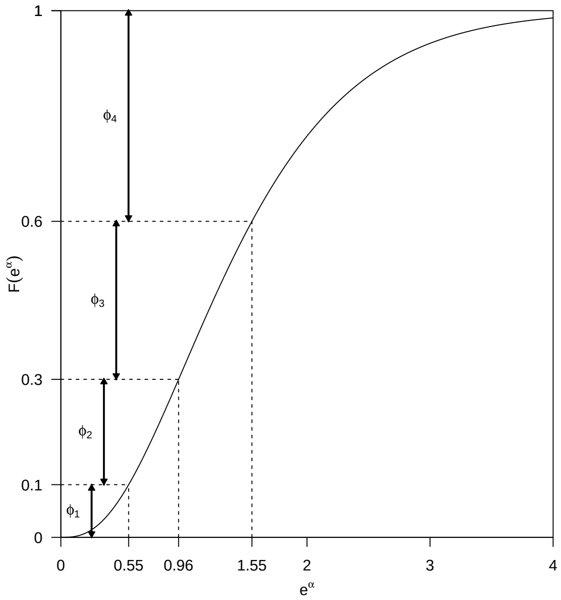

Figure 1. The relationship between the mixture proportions, ϕ_j and the quantities estimated during the optimization procedure, α_p.

To transform from the ϕ_js back to the α_ps we simply re-arrange the above expression.

$$\alpha_p = \log_e \Big(F^{-1}\Big(\phi_j + F(\sum_{p=1}^{j-1} e^{\alpha_p})\Big) - \sum_{p=1}^{j-1} e^{\alpha_p}\Big).$$

Note that we only need as many α_ps as we had ϕ_js, so we do not require any additional parameters.

References

1. Gelman A, Carlin JB, Stern HS, Dunson DB, Vehtari A (2013) Bayesian Data Analysis. CRC Press.

2. Marin JM, Mengersen K, Robert CP (2005) Bayesian modelling and inference on mixtures of distributions. Handbook of statistics 25: 459–507.

3. Press WH, Flannery BP, Teukolsky SA, Vetterling WT (1990) Numerical recipes. Cambridge University Press.

4. Dempster AP, Laird NM, Rubin DB (1977) Maximum likelihood from incomplete data via the EM algorithm. Journal of the Royal Statistical Society Series B, Statistical Methodology .

5. Beavers S, Ramsey F (1998) Detectability analysis in transect surveys. Journal of Wildlife Management : 948–957.
