# Supplementary material for: Mixture Models for Distance Sampling Detection Functions
Source: PLoS One. 2015 Mar 20;10(3):e0118726. doi: 10.1371/journal.pone.0118726 (PMC4368789; doi:10.1371/journal.pone.0118726)
Supplement: S2 Appendix — (PDF) [file pone.0118726.s002.pdf]

Appendix S2: Simulation parameters

David L. Miller[1,*], Len Thomas[1]
1 School of Mathematics and Statistics, and Centre for Research into Ecological and Environmental Modelling, University of St Andrews, St Andrews KY16 9LZ, Scotland
∗ E-mail: dave@ninepointeightone.net

The formulation used for the exponential power series (EPS) detection function in simulation E1 was:

$$g(y, \mathbf{z}; \lambda, b_1) = \exp(-(y/\lambda)^{-b_1})),$$

which has the following pdf:

$$f(y, \mathbf{z}; \lambda, b_1) = \frac{\exp(-(y/\lambda)^{-b_1})),}{\lambda\Gamma(1 + \frac{1}{b_1})}$$

where $\lambda = \exp(\beta_1)$ is a scale parameter and b_1 is a *shape parameter*.

The formulation for the hazard-rate mixture in simulation E2 was:

$$g(y, \mathbf{z}; \boldsymbol{\theta}, \boldsymbol{\phi}) = \sum_{j=1}^{J} \phi_j (1 - \exp(-(y/\sigma_j)^{-b_j})),$$

where b_j is the shape parameter associated with the jth mixture component.

Table 1. Parameters of the detection functions used in the simulations in Section 3 and the true average detection probability (P_a) for each model. Note that for covariate models, β_1 corresponds to the intercept of the first mixture component, β_2 to the intercept of the second mixture component and β_3 to the coefficient for the (common) covariate effect. Numbering is as in Figures 2 and 3 in the main article.

Model	Scenario	β_1	β_2	β_3	π_1	π_2	b_1	b_2	P_a
Line transect	A1	-0.223	-1.897		0.3				0.369
	A2	-0.511	-2.303		0.7				0.514
	A3	2.303	-1.609		0.15				0.363
	A4	-0.357	-2.996		0.6				0.471
Point transect	B1	-0.223	-1.897		0.3				0.24
	B2	-0.511	-2.303		0.7				0.384
	B3	2.303	-1.609		0.15				0.218
	B4	-0.357	-2.996		0.6				0.378
3-point	C1	-0.22	-0.69	-2.3	0.3	0.3			0.505
	C2	2.71	-1.39	-3.0	0.1	0.4			0.257
Covariate	D1	-2.303	-0.288	-0.511	0.4				0.422
	D2	-1.609	-0.223	-0.916	0.4				0.389
EPS	E1	-0.534					1.5		0.5
Hazard-rate	E2	-1.69	-0.304		0.5		7	7	0.5
