# Supplementary material for: Mixture Models for Distance Sampling Detection Functions
Source: PLoS One. 2015 Mar 20;10(3):e0118726. doi: 10.1371/journal.pone.0118726 (PMC4368789; doi:10.1371/journal.pone.0118726)
Supplement: S1 Text — (PDF) [file pone.0118726.s003.pdf]

Text S1: Derivatives of the likelihood

David L. Miller[1,*], Len Thomas[1]
1 School of Mathematics and Statistics, and Centre for Research into Ecological and Environmental Modelling, University of St Andrews, St Andrews KY16 9LZ, Scotland
∗ E-mail: dave@ninepointeightone.net

As mentioned in Appendix S1, maximization is performed on the log-likelihood. In this section we give the derivations of the derivatives of the log-likelihood, which were used to aid optimization. In the main paper we used the symbol y to denote the distance from transect to object. In practice, this distance is a perpendicular distance in the case of line transects and a radial distance for point transects. Here, to avoid confusion, perpendicular distances in the line transect case are denoted by x and radial distances in the point transect case are denoted by r.

Line transects

Starting from the log-likelihood:

$$l(\boldsymbol{\theta}, \boldsymbol{\phi}; \mathbf{x}, \mathbf{Z}) = \sum_{i=1}^{n} \Big(\log \sum_{j=1}^{J} \phi_j g_j(x_i, \mathbf{Z}; \boldsymbol{\theta}_j) - \log \sum_{j=1}^{J} \phi_j \mu_{ij} \Big) \tag{1}$$

we derive the derivatives with respect to the optimisation parameters.

With respect to β_{0j*}

For the intercept terms (also considering in the non-covariate case, these are just the parameters), the parameters have no effect outside of their mixture (ie. β_{0j*} only has an influence on mixture component $j*$), so we can write:

$$\frac{\partial l(\boldsymbol{\theta}, \boldsymbol{\phi}; \mathbf{x}, \mathbf{Z})}{\partial \beta_{0j*}} = \sum_{i=1}^{n} \frac{1}{g(x_i, \mathbf{Z}; \boldsymbol{\theta}, \boldsymbol{\phi})} \phi_{j*} \frac{\partial}{\partial \beta_{0j*}} g_{j*}(x_i, \mathbf{Z}; \boldsymbol{\theta}_{j*}) - \frac{\phi_{j*}}{\mu_i} \frac{\partial}{\partial \beta_{0j*}} \mu_{ij*}.$$

Now, to first find $\frac{\partial}{\partial \beta_{0j*}} g_{j*}(x_i, \mathbf{Z}; \boldsymbol{\theta}_{j*})$:

$$\frac{\partial g_{j*}(x_i, \mathbf{Z}; \boldsymbol{\theta}_{j*})}{\partial \beta_{0j*}} = \frac{\partial}{\partial \beta_{0j*}} \exp\Big(- \frac{x_i^2}{2\sigma_{j*}^2} \Big),$$

applying the chain rule and remembering that σ_{j*} is a (trivial) function of the β_{0j}s:

$$\frac{\partial g_{j*}(x_i, \mathbf{Z}; \boldsymbol{\theta}_{j*})}{\partial \beta_{0j*}} = \Big(\frac{x_i}{\sigma_{j*}} \Big)^2 \exp\Big(- \frac{x_i^2}{2\sigma_{j*}^2} \Big)$$

Expressing μ_{ij*} in terms of the error function, Erf:

$$\begin{aligned}
\frac{\partial \mu_{ij*}}{\partial \beta_{0j*}} &= \frac{\partial}{\partial \beta_{0j*}} \Big(\sqrt{\frac{\pi}{2}} \sigma_{j*} \text{Erf}\Big(\frac{w}{\sqrt{2\sigma_{j*}^2}} \Big) \Big) \\
&= \text{Erf}\Big(\frac{w}{\sqrt{2\sigma_{j*}^2}} \Big) \frac{\partial}{\partial \beta_{0j*}} \Big(\sqrt{\frac{\pi}{2}} \sigma_{j*} \Big) + \sqrt{\frac{\pi}{2}} \sigma_{j*} \frac{\partial}{\partial \beta_{0j*}} \Big(\text{Erf}\Big(\frac{w}{\sqrt{2\sigma_{j*}^2}} \Big) \Big)
\end{aligned} \tag{2}$$

To find $\frac{\partial}{\partial \beta_{0j*}} \mathrm{Erf}\Big(\frac{w}{\sqrt{2\sigma_{j*}^2}}\Big)$, note that we can write and then apply the chain rule:

$$\begin{aligned}\frac{\partial}{\partial \beta_{0j*}} \mathrm{Erf}\Big(\frac{w}{\sqrt{2\sigma_{j*}^2}}\Big) &= \frac{\partial}{\partial \beta_{0j*}} S(u(\sigma_{j*})) \\ &= \frac{\partial S(u)}{\partial u} \frac{\partial u(\sigma_{j*})}{\partial \sigma_{j*}} \frac{\partial \sigma_{j*}}{\partial \beta_{0j*}}\end{aligned}$$

where

$$S(u) = \int_0^u \exp(-t^2)\mathrm{d}t \quad \text{and} \quad u(\sigma_{j*}) = \frac{w}{\sqrt{2\sigma_{j*}^2}}.$$

Their derivatives being

$$\frac{\partial S(u)}{\partial u} = \frac{2}{\sqrt{\pi}} \exp(-u^2), \quad \frac{\partial u(\sigma_{j*})}{\partial \sigma_{j*}} = -\frac{w}{\sqrt{2}} \sigma_{j*}^{-2}.$$

Given these terms, it is just a case of multiplying them:

$$\frac{\partial S(u)}{\partial u} \frac{\partial u(\sigma_{j*})}{\partial \sigma_{j*}} \frac{\partial \sigma_{j*}}{\partial \beta_{0j*}} = -\sqrt{\frac{2}{\pi}} \frac{w}{\sigma_{j*}} \exp\Big(-\frac{w^2}{2\sigma_{j*}^2}\Big)$$

Substituting into (2):

$$\frac{\partial \mu_{ij*}}{\partial \beta_{0j*}} = \mu_{ij*} - w \exp\Big(-\frac{w^2}{2\sigma_{j*}^2}\Big)$$

Finally, the derivative is:

$$\frac{\partial l(\boldsymbol{\theta}, \boldsymbol{\phi}; \mathbf{x}, \mathbf{Z})}{\partial \beta_{0j*}} = \sum_{i=1}^n \Big(\frac{x_i}{\sigma_{j*}}\Big)^2 \phi_{j*} \frac{g_{j*}(x_i, \mathbf{Z}; \boldsymbol{\theta}_{j*})}{g(x_i, \mathbf{Z}; \boldsymbol{\theta}, \boldsymbol{\phi})} - \frac{\phi_{j*}}{\mu_i}(\mu_{ij*} - w g_{j*}(w, \mathbf{Z}; \boldsymbol{\theta}_{j*})).$$

With respect to β_{k*}

Derivatives with respect to the common covariate parameters are found in a similar way to above. The expressions are slightly more complicated since the β_ks effect all of the mixture components.

$$\frac{\partial l(\boldsymbol{\theta}, \boldsymbol{\phi}; \mathbf{x}, \mathbf{Z})}{\partial \beta_{k*}} = \sum_{i=1}^n \Big(\frac{1}{g(x_i, \mathbf{Z}; \boldsymbol{\theta}, \boldsymbol{\phi})} \sum_{j=1}^J \phi_j \frac{\partial}{\partial \beta_{k*}} g_j(x_i, \mathbf{Z}; \boldsymbol{\theta}_j) - \frac{1}{\mu_i} \sum_{j=1}^J \phi_j \frac{\partial}{\partial \beta_{k*}} \mu_{ij}\Big)$$

Every σ_j is a function of the β_ks, so:

$$\begin{aligned}\frac{\partial \sigma_j}{\partial \beta_{k*}} &= \frac{\partial}{\partial \beta_{k*}} \exp\Big(\beta_{0j} + \sum_{k=1}^K z_{ik}\beta_k\Big), \\ &= z_{ik*}\sigma_j.\end{aligned}$$

Hence:

$$\frac{\partial}{\partial \beta_{k*}} \exp\Big(-\frac{x_i^2}{2\sigma_j^2}\Big) = z_{k*}\Big(\frac{x_i}{\sigma_j}\Big)^2 \exp\Big(-\frac{x_i^2}{2\sigma_j^2}\Big) = z_{k*}\Big(\frac{x_i}{\sigma_j}\Big)^2 g_j(x_i, \mathbf{Z}; \boldsymbol{\theta}_j). \tag{3}$$

And so for the μ_{ij}s:

$$\frac{\partial \mu_{ij}}{\partial \beta_{k*}} = z_{ik*}\Big(\mu_{ij} - w \exp\Big(-\frac{w^2}{2\sigma_j^2}\Big)\Big)$$

The derivative is then:

$$\frac{\partial l(\boldsymbol{\theta}, \boldsymbol{\phi}; \mathbf{x}, \mathbf{Z})}{\partial \beta_{k*}} = \sum_{i=1}^{n}\Big(\frac{1}{g(x_i, \mathbf{Z}; \boldsymbol{\theta}, \boldsymbol{\phi})}\sum_{j=1}^{J}\phi_j z_{k*}\Big(\frac{x_i}{\sigma_j}\Big)^2 g_j(x_i, \mathbf{Z}; \boldsymbol{\theta}_j) - \frac{1}{\mu_i}\sum_{j=1}^{J}\phi_j z_{ik*}(\mu_{ij} - w g_j(x_i, \mathbf{Z}; \boldsymbol{\theta}_j))\Big)$$

With respect to α_{j*}

First note that we can write the likelihood (1) as:

$$\begin{aligned} l(\boldsymbol{\theta}, \boldsymbol{\phi}; \mathbf{x}, \mathbf{Z}) = \sum_{i=1}^{n}\Big(&\log\Big(\sum_{j=1}^{J-1}\phi_j g_j(x_i, \mathbf{Z}; \boldsymbol{\theta}_j) + (1 - \sum_{j=1}^{J-1}\phi_j) g_J(x_i, \mathbf{Z}; \boldsymbol{\theta}_J)\Big) \\ &- \log\Big(\sum_{j=1}^{J-1}\phi_j \mu_{ij} + (1 - \sum_{j=1}^{J-1}\phi_j)\mu_{ij}\Big)\Big) \end{aligned}$$

The derivatives with respect to the α_{j*} of this expression are then:

$$\begin{aligned} \frac{\partial l(\boldsymbol{\theta}, \boldsymbol{\phi}; \mathbf{x}, \mathbf{Z})}{\partial \alpha_{j*}} =& \Big(\sum_{i=1}^{n}\frac{1}{g(x_i, \mathbf{Z}; \boldsymbol{\theta}, \boldsymbol{\phi})}\Big(\sum_{j=1}^{J-1} g_j(x_i, \mathbf{Z}; \boldsymbol{\theta}_j)\frac{\partial \phi_j}{\partial \alpha_{j*}} - g_J(x_i, \mathbf{Z}; \boldsymbol{\theta}_J)\sum_{j=1}^{J-1}\frac{\partial \phi_j}{\partial \alpha_{j*}}\Big) \\ &- \frac{1}{\mu_i}\Big(\sum_{j=1}^{J-1}\mu_{ij}\frac{\partial \phi_j}{\partial \alpha_{j*}} - \mu_{iJ}\sum_{j=1}^{J-1}\frac{\partial \phi_j}{\partial \alpha_{j*}}\Big)\Big) \end{aligned} \tag{4}$$

Finding the derivatives is then simply a matter of finding the derivatives of ϕ_j with respect to α_{j*} and substituting them back into (4).

$$\frac{\partial \phi_j}{\partial \alpha_{j*}} = \frac{\partial}{\partial \alpha_{j*}} F(\sum_{p=1}^{j} e^{\alpha_p}) - \frac{\partial}{\partial \alpha_{j*}} F(\sum_{p=1}^{j-1} e^{\alpha_p}).$$

Looking at each of the terms:

$$\frac{\partial}{\partial \alpha_{j*}} F(\sum_{p=1}^{j} e^{\alpha_p}) = A_j = \begin{cases} e^{\alpha_{j*}} f(\sum_{p=1}^{j} e^{\alpha_p}) & \text{for } j \geq j*, \\ 0 & \text{for } j < j*. \end{cases}$$

and

$$\frac{\partial}{\partial \alpha_{j*}} F(\sum_{p=1}^{j-1} e^{\alpha_p}) = A_{(j-1)} = \begin{cases} e^{\alpha_{j*}} f(\sum_{p=1}^{j-1} e^{\alpha_p}) & \text{for } j-1 \geq j*, \\ 0 & \text{for } j-1 < j*. \end{cases}$$

So

$$\frac{\partial \phi_j}{\partial \alpha_{j*}} = A_j - A_{j-1}.$$

Substituting these back into (4) and re-arranging gives:

$$\begin{aligned} \frac{\partial l(\boldsymbol{\theta}, \boldsymbol{\phi}; \mathbf{x}, \mathbf{Z})}{\partial \alpha_{j*}} = \sum_{i=1}^{n}\Big(&\frac{1}{g(x_i, \mathbf{Z}; \boldsymbol{\theta}, \boldsymbol{\phi})}\sum_{j=1}^{J-1}(A_j - A_{j-1})(g_j(x, \mathbf{Z}; \boldsymbol{\theta}_j) - g_J(x, \mathbf{Z}; \boldsymbol{\theta}_J)) \\ &- \frac{1}{\mu_i}\sum_{j=1}^{J-1}(A_j - A_{j-1})(\mu_{ij} - \mu_{iJ})\Big) \end{aligned}$$

Point transects

We now provide the corresponding quantities for point transects, starting from the log-likelihood:

$$l(\boldsymbol{\theta}, \boldsymbol{\phi}; \mathbf{r}, \mathbf{Z}) = n \log 2\pi + \sum_{i=1}^{n} \Big(\log r_i + \log \sum_{j=1}^{J} \phi_j g_j(r_i, \mathbf{Z}; \boldsymbol{\theta}_j) - \log \sum_{j=1}^{J} \phi_j \nu_{ij} \Big). \tag{5}$$

With respect to β_{0j}

From (5), one can see that we obtain:

$$\begin{aligned}
\frac{\partial l(\boldsymbol{\theta}, \boldsymbol{\phi}; \mathbf{r}, \mathbf{Z})}{\partial \beta_{0j*}} &= \sum_{i=1}^{n} \Big(\frac{\partial}{\partial \beta_{0j*}} \log \sum_{j=1}^{J} \phi_j g_j(r_i, \mathbf{Z}; \boldsymbol{\theta}_j) - \frac{\partial}{\partial \beta_{0j*}} \log \sum_{j=1}^{J} \phi_j \nu_{ij} \Big) \\
&= \sum_{i=1}^{n} \Big(\frac{\phi_{j*} \frac{\partial}{\partial \beta_{0j*}} g_{j*}(r_i, \mathbf{Z}; \boldsymbol{\theta}_j)}{g(r_i, \mathbf{Z}; \boldsymbol{\theta}, \boldsymbol{\phi})} - \frac{\phi_{j*} \frac{\partial}{\partial \beta_{0j*}} \nu_{ij*}}{\sum_{j=1}^{J} \phi_j \nu_{ij}} \Big)
\end{aligned}$$

the first part of which (the derivatives of the detection function) are as in the line transect case. The derivatives of ν_{ij} are simpler in the point transect case, since there is an easy analytic expression for ν_{ij} when g_j is half-normal :

$$\nu_{ij} = 2\pi\sigma_{ij}^2 (1 - \exp(-w^2/2\sigma_{ij}^2))$$

then simply applying the product rule yields:

$$\frac{\partial \nu_{ij}}{\partial \beta_{0j*}} = 2(\nu_{ij*} + \pi w^2 g_{j*}(w)).$$

Substituting this into the above expression:

$$\frac{\partial l(\boldsymbol{\theta}, \boldsymbol{\phi}; \mathbf{r}, \mathbf{Z})}{\partial \beta_{0j*}} = \sum_{i=1}^{n} \Big(\frac{\phi_{j*} (r_i/\sigma_{j*})^2 g_{j*}(r_i, \mathbf{Z}; \boldsymbol{\theta}_{j*})}{g(r_i, \mathbf{Z}; \boldsymbol{\theta}, \boldsymbol{\phi})} - \frac{\phi_{j*} 2(\nu_{j*} + \pi w g_{j*}(w))}{\sum_{j=1}^{J} \phi_j \nu_{ij}} \Big)$$

With respect to β_{k*}

Again working from (5), we obtain:

$$\begin{aligned}
\frac{\partial l(\boldsymbol{\theta}, \boldsymbol{\phi}; \mathbf{r}, \mathbf{Z})}{\partial \beta_{k*}} &= \sum_{i=1}^{n} \Big(\frac{\partial}{\partial \beta_{k*}} \log \sum_{j=1}^{J} \phi_j g_j(r_i, \mathbf{Z}; \boldsymbol{\theta}_j) - \frac{\partial}{\partial \beta_{k*}} \log \sum_{j=1}^{J} \phi_j \nu_{ij} \Big) \\
&= \sum_{i=1}^{n} \Big(\frac{\sum_{j=1}^{J} \phi_j \frac{\partial}{\partial \beta_{k*}} g_j(r_i, \mathbf{Z}; \boldsymbol{\theta}_j)}{g(r_i, \mathbf{Z}; \boldsymbol{\theta}, \boldsymbol{\phi})} - \frac{\sum_{j=1}^{J} \phi_j \frac{\partial}{\partial \beta_{k*}} \nu_{ij}}{\sum_{j=1}^{J} \phi_j \nu_{ij}} \Big)
\end{aligned}$$

The derivatives of g_j are as in (3). For ν_{ij}:

$$\frac{\partial \nu_{ij}}{\partial \beta_{k*}} = 2 z_{ik*} (\nu_{ij} - \pi w^2 g_j(w))$$

Putting that together:

$$\frac{\partial l(\boldsymbol{\theta}, \boldsymbol{\phi}; \mathbf{r}, \mathbf{Z})}{\partial \beta_{k*}} = \sum_{i=1}^{n} \Big(\frac{\sum_{j=1}^{J} \phi_j z_{k*} \left(\frac{x_i}{\sigma_j} \right)^2 g_j(x_i, \mathbf{Z}; \boldsymbol{\theta}_j)}{g(r_i, \mathbf{Z}; \boldsymbol{\theta}, \boldsymbol{\phi})} - \frac{\sum_{j=1}^{J} \phi_j 2 z_{ik*} (\nu_{ij} - \pi w^2 g_j(w))}{\sum_{j=1}^{J} \phi_j \nu_{ij}} \Big).$$
