# Supplementary material for: Mixture Models for Distance Sampling Detection Functions
Source: PLoS One. 2015 Mar 20;10(3):e0118726. doi: 10.1371/journal.pone.0118726 (PMC4368789; doi:10.1371/journal.pone.0118726)
Supplement: S2 Text — (PDF) [file pone.0118726.s004.pdf]

Text S2: Variance estimation for mixture model detection functions

David L. Miller[1,*], Len Thomas[1]
1 School of Mathematics and Statistics, and Centre for Research into Ecological and Environmental Modelling, University of St Andrews, St Andrews KY16 9LZ, Scotland
∗ E-mail: dave@ninepointeightone.net

Variances of both $\hat{N}$ and $\hat{P}_a$ can be estimated for both non-covariate models and covariate models using standard methods [1–3].

In the most general case, the variance of $\hat{N}$ is estimated by:

$$\hat{\text{Var}}\left(\hat{N}\right) = \left(\frac{\partial \hat{N}}{\partial \hat{\mathbf{\Theta}}}\right)^{\text{T}} \hat{\mathbf{I}}(\hat{\mathbf{\Theta}})^{-1} \frac{\partial \hat{N}}{\partial \hat{\mathbf{\Theta}}} + \sum_{i=1}^{n} \frac{(1-p_i)}{p_i^2}$$

where $\hat{\mathbf{I}}(\hat{\mathbf{\Theta}})^{-1}$ is the inverse of the Fisher information matrix, $\hat{\mathbf{\Theta}}$ is a vector of all of the maximum likelihood estimates of the parameters of the detection function ($\hat{\mathbf{\Theta}} = (\hat{\boldsymbol{\theta}}, \hat{\boldsymbol{\phi}})$) and all other notation is as in previous sections.

Then for the average detectability:

$$\hat{\text{Var}}\left(\hat{P}_a\right) = \hat{P}_a^2 \left\{ \frac{\hat{\text{Var}}(\hat{N})}{\hat{N}^2} + \frac{\left(\frac{\partial \hat{P}_a}{\partial \hat{\mathbf{\Theta}}}\right)^{\text{T}} \hat{\mathbf{I}}(\hat{\mathbf{\Theta}})^{-1} \frac{\partial \hat{P}_a}{\partial \hat{\mathbf{\Theta}}} + \sum_{i=1}^{n}(1-p_i)}{n^2} - \frac{2\left(\left(\frac{\partial \hat{N}}{\partial \hat{\mathbf{\Theta}}}\right)^{\text{T}} \hat{\mathbf{I}}(\hat{\mathbf{\Theta}})^{-1} \frac{\partial \hat{P}_a}{\partial \hat{\mathbf{\Theta}}} + \sum_{i=1}^{n} \frac{(1-p_i)}{p_i^2}\right)}{n\hat{N}} \right\}.$$

References

1. Borchers DL, Buckland ST, Zucchini W (2002) Estimating Animal Abundance . Springer.

2. Marques F, Buckland ST (2003) Incorporating covariates into standard line transect analyses. Biometrics 59: 924–935.

3. Borchers DL, Buckland ST, Goedhart P, Clarke ED, Hedley SL (1998) Horvitz-Thompson estimators for double-platform line transect surveys. Biometrics : 1221–1237.
